# Comparison of Short-Term Outcomes Between Endovenous 1,940-nm Laser Ablation and Radiofrequency Ablation for Incompetent Saphenous Veins

**DOI:** 10.3389/fsurg.2020.620034

**Published:** 2020-12-11

**Authors:** Insoo Park, Sun-Cheol Park

**Affiliations:** ^1^Charm Vascular Clinic, Seoul, South Korea; ^2^Division of Vascular and Transplant Surgery, Department of Surgery, College of Medicine, The Catholic University of Korea, Seoul, South Korea

**Keywords:** varicose veins, endovenous laser ablation, radiofrequency ablation, RFA, 1940 nm

## Abstract

**Background:** Radiofrequency ablation (RFA) has shown faster recovery and lower pain scores compared to Endovenous laser ablation (EVLA) for treatment of varicose veins. However, a comparison of 1,940-nm EVLA and RFA has not been reported. This study compared short-term outcomes using 1,940-nm EVLA and RFA for varicose veins.

**Methods:** Between April 2018 and June 2018, 43 patients (83 incompetent saphenous veins) were treated with 1,940-nm EVLA and 37 patients (64 incompetent saphenous veins) with RFA. Follow-up duplex was checked at 1 month and 3 months.

**Results:** Baseline characteristics showed no significant differences between both groups except for age. Pain scores at 6 h, and at 1, 10, and 30 days after treatment showed no differences. Complications and time to return to normal activity showed no differences. The 100% closure rate was checked in both groups at 1 month and 3 months follow-up.

**Conclusion:** Short-term outcomes showed no significant differences between 1,940-nm EVLA and RFA treatment.

## Introduction

In the past several decades, treatment of varicose veins has undergone substantial changes, including a transition from surgical stripping to endothermal ablation (1–3). Endothermal ablation is now recommended as first-line treatment due to rapid recovery and fewer side effects compared to surgical stripping (4, 5).

Two classic types of endothermal ablation are endovenous laser ablation (EVLA) and radiofrequency ablation (RFA). Both modalities perform endothermal ablation but differ in their mechanism and technique (6, 7).

Both modalities produce good outcomes, but comparative studies reported that RFA showed less postoperative pain and quicker recovery time than ELVA (6–10). However, all of these studies compared RFA with 810-nm or 980-nm EVLA, which were lower wavelengths. The wavelength of lasers used for EVLA has changed over time. Lower wavelengths (810, 940, and 980 nm) were generally used in the past, and higher wavelengths (1,320, 1,470, and 1,940 nm) have become popularized in recent years. Lower wavelengths are associated with higher hemoglobin absorption and higher wavelengths are associated with higher water absorption (11, 12). Since introduction of the 1,470-nm laser, the proportion of water absorption has increased, enabling effective treatment even at lower power (W) and linear endovenous energy density (LEED, J/cm), thereby leading to less thermal damage (13, 14).

Therefore, recent studies reported that using a 1,470-nm laser led to the same or even less postoperative pain and bruising compared to that associated with RFA (15–18).

However, to the best of our knowledge, no study has compared RFA with EVLA using a wavelength of 1,940 nm, the highest currently available. We report the retrospectively comparative results of short-term outcomes using RFA treatment and 1940-nm diode laser treatment.

## Materials and Methods

### Patients

A total of 115 patients who underwent RFA or EVLA at the Charm Vascular Clinic between April 2018 and July 2018 were enrolled in this study. All patients were provided an explanation of the study and gave informed consent. Ethical approval was gained from the Institutional Review Board of The Catholic University of Korea, Seoul St. Mary's Hospital (No. KC19RESI0138). This is an observational prospective study.

All targeted veins for treatment demonstrated at least 0.5 s of reflux and a diameter of ≥3 mm at 3 cm distal to the saphenofemoral junction (SFJ) in standing position.

The exclusion criteria were as follows: (1) diameter of the treated vein > 12 mm, (2) length of epifascial saphenous vein > 10 cm, (3) number of concomitant phlebectomies > 20, (4) recurrent varicose vein, or (5) patient refusal to participate. Thirty-five patients were excluded, resulting in 80 patients included in the study.

The preoperative Clinical, Etiologic, Anatomic, Pathophysiologic (CEAP) grades was C1–C5 (C1: telangiectasia or reticular veins, C2: varicose veins, C3: edema, C4a: pigmentation or eczema, C4b: lipodermatosclerosis or atrophie blanche, C5: healed ulcer). We classified the patients as per highest clinical grade of the two limbs. Three patients with C1 grade were included due to symptoms such as aching, cramping, heaviness, and tingling.

The Revised Venous Clinical Severity Score (rVCSS) and Aberdeen Varicose Vein Questionnaires (AVVQ) were determined before treatment. We determined patients as per highest rVCSS of the two limbs. Post-treatment pain was assessed with the numerical pain rating scale (NRS; 0–10), and the number of days until return to normal activity was determined. Duplex sonograms were performed at 1 month and 3 months after treatment. rVCSS and AVVQ score were checked at 3 months after treatment. All procedures were performed by one surgeon who has treated >500 cases each with EVLA and RFA.

### Procedures

On the day of treatment, vein locations were mapped on the patient's leg in standing position using sonography (P7, GE, Milwaukee, Wisconsin, USA). Treatment was performed under light intravenous sedation using propofol or midazolam under supervision of an anesthesiologist. All great saphenous vein (GSV) access was attained through a puncture between knee joint and mid-calf level, with no ankle level puncture. During ablation, 30° Trendelenburg position was used. For GSV treatment, ablation was initiated from 2 cm distal to SFJ but not maintained below the knee area. For small saphenous vein (SSV) access, puncture was performed at the lower calf. Ablation was initiated from 2 cm distal to sapheno-popliteal junction (SPJ), however, when the SPJ was not clear, ablation was initiated from the saphenous fascial curve, but not maintained below the low calf area.

Tumescent solution was prepared by mixing 500 cc of Hartman solution with 20 cc of 2% lidocaine, and perivenous infiltration was performed using a motor pump while monitoring the intraoperative sonogram (LOGIQe, GE, Milwaukee, Wisconsin, USA). For epifascial compartment of GSV, ablation was performed after adequate perivenous infiltration to at least 10 mm away from the skin.

### EVLA

For EVLA, access was gained using a 16-G angio needle, and a ball-tip fiber (CareTech, Sung-nam, Korea) was directly inserted into the 16-G needle without using an introducer sheath or guide. An about 5 cm length of initial ablation area was manually compressed during ablation, and compression was not applied below this area. Power and manual pull-back speed were mostly set to 6 W and 0.10–0.15 cm/sec, and did not get out of the 3–6 W and 0.1–0.2 cm/sec ranges. Speed for the proximal area near the junction was maintained at 0.10 cm/s; when vein diameter decreased as the fiber moved downward, the pull-back speed was elevated at the surgeon's discretion.

### RFA

RFA was performed using the ClosureFast catheter (VNUS Medical Technologies, San Jose, CA, USA) according to the manufacturer's instructions. Access was gained using a 7-Fr introducer sheath (Terumo, RADIOFOCUS INTRODUCER II Fr. 7, Japan). The first proximal segment was treated with double-cycle ablation, and single-cycle ablation was applied to areas below this segment. Manual compression was performed over the veins throughout the ablation.

After completing the above both procedures, concomitant phlebectomy or sclerotherapy was performed for a branching varix or reticular vein/telangiectasia at the surgeon's discretion. Patients were discharged on the same day, about 4–8 h after the procedure, and were advised to ambulate. As post-procedural medication, oral non-steroidal anti-inflammatory drugs were taken for 3 days, and patients were advised to wear thigh-level 20–30-mmHg compressive stockings for 2 weeks during the day time. Thromboprophylaxis medications were not prescribed. Pain score (NRS) was measured at 6 h, and at 1, 10, and 30 days after procedure. The number of days until return to normal activity was surveyed over the phone at 1 week after treatment. Sonogram follow-up was performed at 1 month and 3 months after treatment. Complications were assessed during the 1-month follow-up period.

### Statistical Analysis

All data are presented as mean + standard deviation. A two-tailed Student *t*-test was used to calculate statistical significance for continuous variables. All statistical analyses were performed using SPSS version 18.0 (IBM, Armonk, NY, USA). Statistical significance was assumed at a *p*-value < 0.05.

## Results

Tables 1, 2 show patient demographics and treatment characteristics. With the exception of age, the two groups did not differ in any characteristics. Time for procedure was 3 min longer on average with the EVLA the difference was not statistically significant.

**Table 1:** Patient demographics mean (±SD), range, or number (%).

	**EVLA (*n* = 43)**	**RFA (*n* = 37)**	***p*-value**
Female	30 (69.8%)	25 (67.6%)	0.227
Age	47.4 ± 13.8	40.4 ± 14.3	0.030
BMI	22.7 ± 3.4	23.2 ± 3.4	0.539
CEAP			0.609
C1	2	1	
C2	19	17	
C3	19	16	
C4	3	3	
C5	–	1	
No of treated veins/patient	1.95 ± 0.75	1.76 ± 0.79	0.262
Initial rVCSS	5.0 ± 1.4	4.7 ± 1.7	0.365
Initial AVVQ	14.8 ± 7.6	12.7 ± 5.9	0.193

*EVLA, endovenous laser ablation; RFA, radiofrequency ablation; BMI, body mass index (kg/m^2^); CEAP, Clinical, Etiologic, Anatomic, Pathophysiologic class; rVCSS, Revised Venous Clinical Severity Score; AVVQ, Aberdeen Varicose Vein Questionnaires*.

**Table 2:** Treatment characteristics, mean (±SD).

	**EVLA (*n* = 83)**	**RFA (*n* = 64)**	***p*-value**
Number of treated veins (GSV/SSV)	83 (67/16)	64 (48/16)	
Diameter of vein (cm)	0.50 ± 0.16	0.55 ± 0.19	0.104
Length of vein (cm)	33.75 ± 11.21	35.39 ± 12.19	0.397
Time of procedure (min)	23.51 ± 9.42	20.27 ± 10.79	0.160
No. of concomitant phlebectomy	8.04 ± 5.52	8.59 ± 8.39	0.770
LEED (J/cm)	46.4 ± 9.2	–	

*GSV, great saphenous vein; SSV, small saphenous vein; LEED, linear endovenous energy density (LEED, J/cm)*.

The post-procedural pain score was not significantly different between the two groups at all time points, and the mean LEED (J/cm) for EVLA was 46.4. Return to normal activity (days) was not significantly different between the two groups (Table 3).

**Table 3:** Post-procedure characteristics, mean (±SD).

	**EVLA (*n* = 43)**	**RFA (*n* = 37)**	***p*-value**
Pain score (0~10)			
6 h	1.64 ± 1.31	1.32 ± 1.17	0.261
1 day	0.70 ± 0.91	0.66 ± 1.08	0.835
10 days	0.56 ± 0.86	0.39 ± 0.72	0.313
30 days	0.18 ± 0.38	0.38 ± 0.84	0.208
Return to normal activity (days)	1.16 ± 0.48	1.20 ± 0.53	0.750

The two groups did not significantly differ in complications; there was one case of endovenous heat-induced thrombosis (EHIT) in the EVLA group (Table 4). Which was Kabnick classification 1 in SSV.

**Table 4:** Complication, mean (±SD), range, or number (%).

	**EVLA (*n* = 83)**	**RFA (*n* = 64)**	***p*-value**
Infection	–	–	–
Hyperpigmentation	1	1	0.853
Thrombophlebitis	4	2	0.607
Paresthesia	2	2	0.792
EHIT/DVT	1	–	0.378

*Thrombophlebitis, defined for treated main trunk, not for tributaries; EHIT, endovenous heat induced thrombosis (Kabnick classification); DVT, deep vein thrombosis*.

Duplex sonograms performed 1 month and 3 months after the procedure confirmed that both groups were 100% reflux free rate (Table 5).

**Table 5:** Duplex sonogram at 1 month and 3 months.

	**EVLA (*****n*** **=** **83)**		**RFA (*****n*** **=** **64)**		***p*-value**
	**1 month**	**3 months**	**1 month**	**3 months**	
Follow-up rate	79/83 (95.2%)	49/83 (59.0%)	63/64 (98.4%)	30/64 (46.9%)	–
Anatomical success rate^*^	79/79 (100%)	49/49 (100%)	62/63^a^ (98.4%)	30/30 (100%)	0.261
Reflux free rate^†^	79/79 (100%)	49/49 (100%)	63/63 (100%)	30/30 (100%)	–

* *Definition of anatomical success: lack of flow <3 cm throughout the treated area by duplex imaging. 1 GSV showed color flow without reflux after RFA at 1 month*.

† *Definition of reflux: any reflux flow in treated area > 3 cm*.

Both rVCSS and AVVQ showed statistical significant improvement at 3 months after the treatment. And there was no significant difference between the two groups.

## Discussion

In the present study, the 1,940-nm EVLA group and RFA group did not significantly differ in postoperative pain, recovery time, complications, 1 month and 3 months closure rate.

According to previous literatures, the required energy for good outcomes is about 80–100 LEED (J/cm) with the 1,470-nm EVLA (11, 13, 14). And to date, a few studies have investigated EVLA using the 1,940-nm laser, and this treatment has generally produced good outcomes, even with energy <50 LEED (J/cm) (19–24).

Absorption at particular chromophore and absorption coefficients differ according to laser wavelength. At lower wavelengths (800–900 nm), the absorption coefficient for hemoglobin was higher than that for water. On the other hand, with introduction of water-specific, higher wavelengths, the chromophore changed from hemoglobin to water in vein walls. Thus, it is now theoretically possible to efficiently treat vein walls with lower power than before, with lasers of higher wavelengths (11, 12).

In the 2000s, when the ClosureFast system was introduced, studies found that RFA caused less postoperative pain and bruising with quicker recovery compared to treatment using lower wavelengths of 810 or 980 nm (6–10). However, since the introduction of laser of a higher wavelength, recent several studies, including a prospective 5 years study, reported that 1,470-nm EVLA using a radial fiber was associated with similar outcomes or even less postoperative pain and shorter recovery time compared to RFA (15–18).

We used a ball-tip fiber in this study because radial fibers produced in Korea had some shortcomings during the study period. Using a radial fiber reduces perforation caused by direct contact and leads to efficient circumferential thermal damage, which enables effective treatment at lower power. Thus, we could predict that post-procedural pain would be less than when a bare-tip fiber or ball-tip fiber was used (6). In the future, we will expect to report outcomes with 1,940-nm EVLA when a radial fiber is used.

This study was limited by the non-randomized, single-center setting and relatively small sample of subjects. Moreover, only the short-term post-procedural results were analyzed. Further investigation should evaluate this in future.

## Conclusion

This study is the first comparative study between 1,940-nm EVLA and RFA. And the short-term outcomes between both groups showed no difference in pain score, recovery time, complications, 1 month and 3 months closure rate. Larger and more long-term randomized studies are needed.

## Data Availability Statement

The original contributions generated for this study are included in the article/supplementary materials, further inquiries can be directed to the corresponding author/s.

## Ethics Statement

The studies involving human participants were reviewed and approved by The Institutional Review Board of The Catholic University of Korea, Seoul St. Mary's Hospital. The patients/participants provided their written informed consent to participate in this study.

## Author Contributions

S-CP: study conception. IP: data collection and writing. IP and S-CP: analysis, investigation, critical review, and revision. All authors contributed to the article and approved the submitted version.

## Conflict of Interest

The authors declare that the research was conducted in the absence of any commercial or financial relationships that could be construed as a potential conflict of interest.
